# Afatinib as first-line treatment for advanced lung squamous cell carcinoma harboring uncommon EGFR G719C and S768I co-mutation: A case report and literature review

**DOI:** 10.1016/j.heliyon.2024.e35304

**Published:** 2024-07-26

**Authors:** Ruoyu Deng, Wen Zhang, Jialing Lv, Fang Wang, Yanqiong Chen, Chengqi Jiang, Yaling Guan, Chao Zhang

**Affiliations:** a Department of Oncology, Qujing First People's Hospital/The Qujing Affiliated Hospital of Kunming Medical University, Qujing, 655000, China; b Department of Pathology, Second People's Hospital of Qujing City, Qujing, 655000, China

**Keywords:** Afatinib, Squamous cell carcinoma, Non-small cell lung cancer, Epidermal growth factor receptor, Tyrosine kinase inhibitors

## Abstract

Ten percent of non-small cell lung cancer patients with epidermal growth factor receptor (EGFR) mutations harbor uncommon variants. These mutations are mainly involved in lung adenocarcinomas but are rare in lung squamous cell carcinoma (LSCC). In 2018, the Food and Drug Administration-approved afatinib for this specific patient population. However, there is limited information regarding the effectiveness of afatinib for LSCC with EGFR mutations. This case report documented a unique case of a patient with LSCC, which had a rare compound EGFR mutation (G719C and S768I) and showed significant response to afatinib treatment, with 10 months of progression-free survival. New NTRK1 and RET gene mutations may play a potential role in the development of acquired resistance to afatinib following clinical progression. This case highlights the importance of genetic profiling in patients with LSCC. Although these patients have a low positive rate of EGFR mutations, searching for EGFR mutations in these patients might broaden their treatment options.

## Key points

1. A patient diagnosed with lung squamous cell carcinoma exhibiting rare concurrent epidermal growth factor receptor mutation (G719C and S768I) showed favorable response to afatinib, achieving 10 months of progression-free survival.
2. Next-generation sequencing analysis identified new NTRK1 and RET gene mutations after disease progression.

## 1 Introduction

Patients with non-small cell lung cancer (NSCLC) have a high prevalence of epidermal growth factor receptor (EGFR) mutations, with a frequency of 30%–60 % in Asia [1]. These mutations are mainly involved in lung adenocarcinomas (LUAD) but are rare in lung squamous cell carcinoma (LSCC). The prevalence of EGFR mutations in patients with LSCC is estimated to be 4.2%–7% [2,3]. Among NSCLC patients with EGFR mutations, common mutations make up 75%–80 % of cases [[4], [5], [6]], whereas uncommon mutations, including Ser768Ile (S768I), Leu861Gln (L861Q), and Gly719Xaa (G719X), constitute approximately 10 % [7].

Currently, EGFR tyrosine kinase inhibitors (EGFR-TKIs) are considered as the standard first-line treatment for patients with locally advanced or metastatic NSCLC harboring sensitizing EGFR mutations [8,9]. Afatinib, an oral, second-generation, irreversible EGFR-TKI, selectively inhibits sensitizing and uncommon EGFR mutations [10]. A previous study showed that afatinib exhibits superior clinical activity in NSCLC patients with advanced disease harboring uncommon EGFR mutations, including G719X, L861Q, and S768I [11]. This case report presents the first instance of a patient with advanced LSCC harboring a compound, uncommon EGFR mutation (G719C and S768I) who showed significant response from afatinib treatment.

## 2 Case presentation

**Fig. 1:**
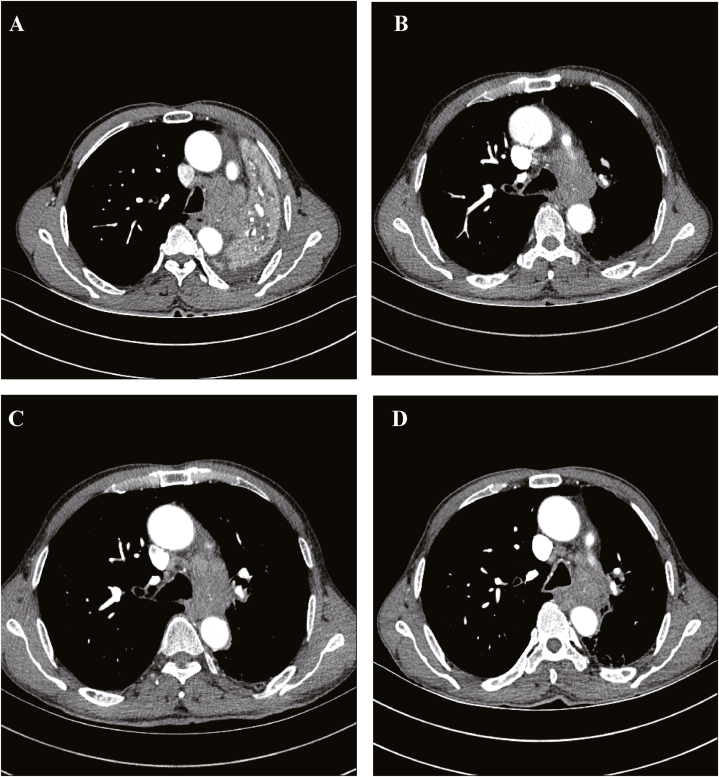
Lung changes on computed tomography (CT) during afatinib treatment. (A) Pretreatment. (B) CT scan at two months after afatinib treatment initiation. (C) CT scan at five months after afatinib treatment initiation. (D) CT scan at eight months after afatinib treatment initiation.

**Fig. 2:**
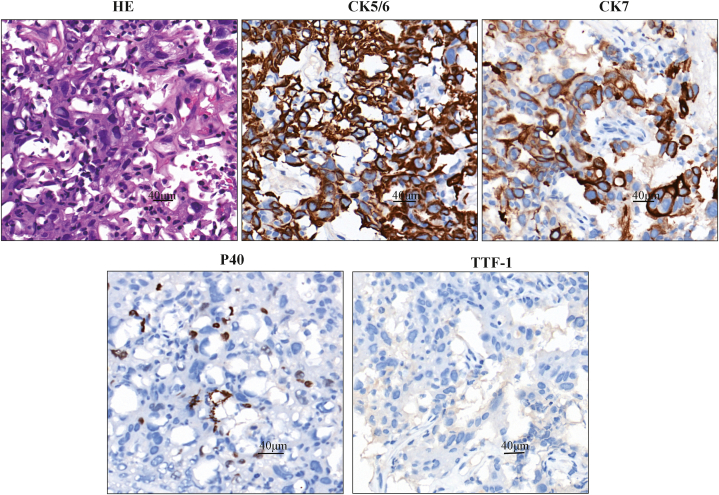
Pathological staining of the primary tumor. Hematoxylin–eosin (H&E) staining. Immunohistochemistry staining positive for CK5/6, CK7, and P40 and negative for TTF1.

**Fig. 3:**
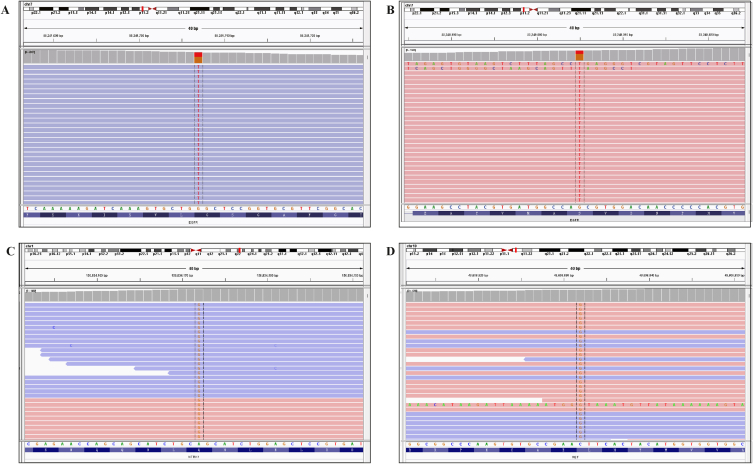
First next-generation sequencing (NGS) results revealing the presence of (A) G719C mutation and (B) S768I mutation. Second NGS results showing the presence of G719C, S768I, (C) NTRK1, and (D) RET mutations.

A 66-year-old male patient with smoking history presented to our hospital in June 2022 with symptoms of cough, sputum, and hemoptysis. An enhanced chest computed tomography (CT) scan revealed a space-occupying lesion at the left lung hilum, causing obstructive pneumonia, along with enlarged left hilar and mediastinal lymph nodes (Fig. 1A). After obtaining informed consent, a biopsy of the lesion in the superior lobe was performed via bronchoscopy. Based on the results, a pathological diagnosis of LSCC was made. Hematoxylin–eosin-stained histological slides showed a nest-like distribution of tumor tissue, with unclear boundaries between cancer cells and visible tumor giant cells. The cytoplasm was abundant and eosinophilic. The size of the nucleus was varied, the nuclear membrane was irregular and polygonal, and the nuclear–cytoplasmic ratio was increased. Immunohistochemistry analysis of the biopsied tissue revealed positive staining for P40, CK5/6, and CK7 and a significant proliferation index showing that 50 % of cells expressed Ki-67. Negative staining results were obtained for napsin A, synaptophysin, thyroid transcription factor 1 (TTF1), chromogranin A, P63, vimentin, and Wilms' tumor-1, whereas positive staining results were obtained for INI-1 and Brg-1 (Fig. 2). Formalin-fixed paraffin-embedded (FFPE) samples with an 80 % tumor cell percentage were prepared from the patient's left lung biopsy tissue under bronchoscopy for next-generation sequencing (NGS) based on targeted DNA. NGS identified an EGFR exon 18 G719C mutation with an allele frequency of 10.58 % and an EGFR exon 20 S768I mutation with an allele frequency of 12.27 % (Fig. 3A and **B**). No additional mutations were found in other key driver genes. The expression of programmed death ligand 1 was less than 1 %. The patient's clinical disease was classified as stage IVA (cT4N2M1a), according to the 8th Edition of the TNM Classification of Malignant Tumors.

The patient began afatinib treatment at a dose of 40 mg daily on August 15, 2022. After 20 days, he developed grade 2 diarrhea based on the Criteria for Adverse Events Version 4.0, prompting a reduction in the dose of afatinib to 30 mg/day because of its impact on the patient's quality of life. Two months post-treatment initiation, a follow-up CT scan revealed a significant decrease in the primary tumor size, lymph node swelling, and obstructive atelectasis, resulting in a partial response (PR) according to the Response Evaluation Criteria in Solid Tumors Version 1.1 (Fig. 1B). Afatinib treatment was continued for 10 months (Fig. 1C and **D**) until a chest CT scan on June 20, 2023 showed rapid disease progression. Subsequent biopsy during repeat bronchoscopy confirmed squamous carcinoma. Thereafter, FFPE samples with 60 % tumor cell percentage were prepared using the patient's left lung biopsy tissue for targeted DNA-based NGS. NGS profiling revealed new mutations in NTRK1 (Q80R) and RET (L481V) alongside existing EGFR mutations (Fig. 3C and **D**). The patient then underwent docetaxel combined with nedaplatin chemotherapy, leading to a PR after four cycles of treatment despite experiencing mild side effects, including grade 1 nausea and grade 1 decrease in white blood cell and neutrophil counts. No long-term adverse effects were reported, and the treatment is currently ongoing.

## 3 Discussion

The emergence of NGS for detecting cancer mutations has facilitated the identification of cancer mutations with greater accuracy and efficiency. However, the frequency of EGFR mutations in LSCC is relatively low, ranging from 4.2 % to 7 %, which is significantly lower than that observed in patients with LUAD [2,3]. The National Comprehensive Cancer Network (NCCN) guidelines advocate for EGFR gene mutation testing in patients with LSCC. Although TKIs are the standard first-line treatment for patients with locally advanced or metastatic NSCLC carrying sensitizing EGFR mutations, their efficacy in treating LSCC remains a topic of debate. In their study on the effectiveness of EGFR-TKIs in LSCC patients with EGFR mutations, Fang et al. [12] found that patients with LSCC had a low frequency of EGFR mutations. Despite the limited effectiveness of EGFR-TKIs in LSCC patients with EGFR mutations, some patients benefited from this treatment. A meta-analysis showed that EGFR-TKIs improved overall survival (OS) and progression-free survival (PFS) compared with placebo in unselected patients with advanced LSCC [13]. In their study including 110 patients with LSCC who were treated with first-generation EGFR-TKIs, Si et al. [14] found that patients with EGFR mutations had longer PFS but similar OS compared to patients with wild-type EGFR. This finding indicates that although EGFR-TKIs are less effective in EGFR-mutant LSCC compared with LUAD, they still confer clinical benefits. Another retrospective study involving 102 patients with LSCC treated with gefitinib or erlotinib revealed that patients with EGFR mutations had longer PFS than those with wild-type EGFR [15]. In conclusion, while the efficacy of EGFR-TKIs in EGFR-mutant LSCC may not be as pronounced as in other types of lung cancers, they still lead to improved outcomes compared with chemotherapy. These findings guided our decision to opt for TKIs over chemotherapy as the first-line treatment of advanced LSCC in this particular case.

Compound mutations, usually containing common and rare mutations, are found in 2%–25 % of EGFR mutation-positive lung cancers [16]. These mutations, especially when involving dual rate mutations, are rare. Moreover, there is limited information regarding the effectiveness of EGFR-TKIs for such cases. Preclinical evidence suggests that NSCLC patients with uncommon compound EGFR mutations exhibit varying sensitivity to different TKIs [17]. Chiu et al. [18] found that patients with uncommon compound EGFR mutations (G719X + L861Q and G719X + S768I) treated with EGFR-TKIs had longer PFS than those with single mutations, with a median PFS of 11.9 months versus 6.5 months (p = 0.010). Afatinib, which is an irreversible inhibitor targeting the entire ERBB family, has shown superior efficacy in patients with EGFR G719X, L861Q, and S768I mutations, as observed in a combined analysis of the LUX-Lung 2, 3, and 6 studies [11]. Consequently, it became the only Food and Drug Administration-approved EGFR-TKI for this specific patient subgroup. A primary analysis of 693 patients with rare EGFR mutations treated in randomized trials supported the use of afatinib, particularly in those with compound mutations [19]. Additionally, a study in Taiwan suggested that patients with uncommon mutations treated with afatinib as first-line therapy had longer PFS than those treated with gefitinib or erlotinib [20]. Despite the demonstrated efficacy of afatinib in LUAD patients with uncommon compound EGFR mutations, further studies are needed to assess its therapeutic potential in LSCC, particularly in cases with compound mutations.

In fact, reports of afatinib use in LSCC patients with EGFR mutation are extremely rare. Han et al. [21] found that a patient with LSCC harboring uncommon compound EGFR mutation (G719A and R776C) benefited from afatinib and achieved PFS of 11 months. Jiang et al. [22] reported for the first time the benefits of combining afatinib with palbociclib in patients with advanced LSCC carrying EGFR L861Q and CDK4 amplification. Matsumura et al. [23] found that a patient with advanced LSCC carrying EGFR 19 del attained partial remission of approximately 1 year after second-line use of afatinib. PR was observed in each of these cases, with an average afatinib treatment duration exceeding 10 months. Here, we present a unique case of a patient with LSCC harboring uncommon compound mutations G719C and S768I within the EGFR gene, a previously unreported event.

EGFR-TKIs exhibit well-defined adverse event (AE) profiles consistent with their mechanism of action. Common treatment-related AEs (TRAEs) associated with these drugs include diarrhea, rash/acne, stomatitis, and effects on the nails. These AEs are typically predictable and can be effectively managed through established protocols for dose modification (afatinib and erlotinib) or interruption (gefitinib) based on individual tolerability [24]. In their analysis using data from the LUX-Lung 3, 6, and 7 studies, Tu et al. [25] found that tolerability-guided dose reduction of afatinib in Chinese patients did not affect median PFS or OS while reducing the incidence of TRAEs. This study supports the practice of initiating patients on afatinib at 40 mg/day and adjusting the dosage as necessary based on tolerability to ensure optimal efficacy. In the specific case presented, the desired therapeutic effect was still achieved even though the dose was reduced because of intolerable side effects.

Real-world data on the benefits and resistance mechanisms of afatinib in patients with uncommon EGFR mutations remain limited. Pang et al. [26] were the first to investigate the spectrum of potential resistance mechanisms in this patient population. Among 27 patients with EGFR G719X/L861Q/S768I mutations who received afatinib treatment, NGS identified potential resistance mutations in 16 patients (59.3 %), including EGFR-T790M (three patients, 11.1 %), CDK4 amplification (three patients, 11.1 %), FGFR1 amplification (three patients, 11.1 %), PIK3CA mutation (three patients, 11.1 %), MET amplification (three patients, 11.1 %), RET fusion (one patient, 3.7 %), HER2 mutation (one patient, 3.7 %), and BRAF mutation (one patient, 3.7 %). In their analysis of resistance mechanisms in a larger cohort of 53 patients with uncommon EGFR mutations, Qin et al. [27] found that MET amplification was the most common acquired resistance mechanism, followed by EGFR T790M. This differs significantly from the resistance mechanisms observed in patients with common EGFR mutations. Additionally, Han et al. [21] reported a case suggesting MYC amplification as a potential contributor to afatinib resistance. In our case, the emergence of resistance to afatinib mutations in NTRK1 and RET genes was identified through NSG.

NTRK1 was initially identified as a fusion oncogene in colorectal cancer in 1986, known as trk A (tropomyosin receptor kinase) [28]. The NTRK gene, which belongs to the neurotrophic tyrosine receptor kinase (NTRK) family alongside NTRK2 and NTRK3, was recognized as the ninth key driver gene in NSCLC according to the 2019 NCCN Guidelines. Recently, NTRK fusion genes have emerged as promising targets for NSCLC treatment. In a study focusing on patients with lung cancer in Northeast China, the mutation rate of the NTRK gene was approximately 4.6 % [29]. In another study, Marchetti et al. [30] found that large cell neuroendocrine carcinoma of the lung does not exhibit mutations in NTRK1, but rather in NTRK2 and NTRK3. Despite the lower reported mutation frequency of the NTRK1 gene compared with fusions, several studies suggest its potential association with tumor development or resistance to TKI treatment [31,32]. Greco et al. [33] reported that the Gly571Arg mutation in NTRK1 can lead to neural receptor inactivation, which leads to autonomic and sensory disorders, ultimately causing congenital insensitivity to pain with anhidrosis. In our case, the patient developed resistance to afatinib treatment, and a Q80R mutation was identified in the NTRK1 gene. Further studies are needed to validate whether this mutation is linked to afatinib resistance.

Approximately 2 % of human cancers exhibit alterations in the receptor tyrosine kinase gene RET, including point mutations or fusions that increase kinase activity and promote tumor cell growth [34]. Activating rearrangements of RET (rearranged during transfection) have been identified as oncogenic factors in several malignancies, including papillary thyroid carcinoma and NSCLC [35]. Pralsetinib (BLU-667) and selpercatinib (LOXO-292, LY3527723), which are two highly potent and selective RET protein TKIs, have received regulatory approval [36,37]. A study using NGS sequencing of 4871 tumors showed that 88 patients had RET abnormalities, with point mutations being the most common type (38.6 %), followed by fusions (30.7 %) [38]. To date, more than 60 activating RET mutations have been reported, some of which are associated with drug resistance, including S904F, V1788N, V804L, G801A, G810S, and G810R [35]. In our case, NGS sequencing detected the L481V mutation after the patient developed afatinib resistance. Whether this is related to afatinib resistance and disease progression remains to be fully elucidated.

Currently, no approved targeted therapies for NTRK1 and RET mutations are available. Physicians typically follow the NCCN guidelines for treatment when patients with EGFR-TKI-resistant disease and NTRK1/RET mutations are identified. These guidelines recommend osimertinib and chemotherapy for T790M-positive and negative tumors, respectively. In this case, the patient with progressive disease received chemotherapy. As of now, the patient remains in good condition.

In conclusion, we report the first case of a patient with LSCC harboring an EGFR G719C/S768I co-mutation who initially benefited from afatinib treatment. New NTRK1 and RET gene mutations may play a potential role in the development of acquired resistance to afatinib. Further studies are needed to validate these findings and gain deeper insights into the resistance mechanisms of afatinib. Additionally, it is important to actively search for the driver gene, even in LSCC cases, in order to broaden treatment options.

## Funding

This work was supported by Ye Xin Expert Workstation of Yunnan Province (No. 202305AF15009).

## Ethics statement

The patient provided informed consent to participate in the study. The patient provided informed consent for the publication of their anonymised case details and images.

## CRediT authorship contribution statement

**Ruoyu Deng:** Writing – original draft. **Wen Zhang:** Writing – review & editing. **Jialing Lv:** Formal analysis. **Fang Wang:** Writing – original draft. **Yanqiong Chen:** Project administration. **Chengqi Jiang:** Data curation. **Yaling Guan:** Writing – review & editing. **Chao Zhang:** Project administration.

## Declaration of competing interest

The authors declare that they have no known competing financial interests or personal relationships that could have appeared to influence the work reported in this paper.
